# Does Historical Coexistence with Dingoes Explain Current Avoidance of Domestic Dogs? Island Bandicoots Are Naïve to Dogs, unlike Their Mainland Counterparts

**DOI:** 10.1371/journal.pone.0161447

**Published:** 2016-09-07

**Authors:** Anke S. K. Frank, Alexandra J. R. Carthey, Peter B. Banks

**Affiliations:** 1 School of Biological Sciences, University of Tasmania, TAS 7001, Tasmania, Australia; 2 Botanical Institute, University of Cologne, Cologne, Germany; 3 Department of Biological Sciences, Macquarie University, North Ryde, NSW 2109, Australia; 4 School of Life and Environmental Sciences, The University of Sydney, NSW 2006, Sydney, Australia; University of Southern Queensland, AUSTRALIA

## Abstract

Introduced predators have a global reputation for causing declines and extinctions of native species. Native prey naiveté towards novel predators is thought to be a key reason for predator impacts. However, naiveté is not necessarily forever: where coexistence establishes, it is likely that naiveté will be reduced through adaptation, and the once alien predator will eventually become recognised by prey. For example, native marsupial bandicoots in Sydney avoid backyards with domestic dogs (*C*. *lupus familiaris*), but not domestic cats (*Felis catus*), even though cats and dogs were both introduced about 200 years ago (Carthey and Banks 2012). The authors attributed bandicoots’ recognition of dogs to long-term exposure to a close relative of dogs, dingoes that arrived in Australia 4000 years ago. Here, we test a prediction of this hypothesis by taking the study to Tasmania, where dingoes have never been present but where domestic dogs also arrived about 200 years ago. We use a similar survey design to that of Carthey and Banks (2012): asking Hobart residents to report on pet-ownership, bandicoot sightings and scats within their backyards, as well as an array of yard characteristic control variables. We predicted that if long term experience with dingoes enabled mainland bandicoots to recognise domestic dogs, then Tasmanian bandicoots, which are inexperienced with dingoes, would not recognise domestic dogs. Our results indicate that Tasmanian bandicoots are naïve to both dogs and cats after only 200 years of coexistence, supporting our hypothesis and the notion that naiveté in native prey towards alien predators (as observed on the mainland) may eventually be overcome.

## Introduction

Worldwide, introduced predators have had a significant effect on native wildlife [1]. Their exaggerated impact in comparison to native predators is commonly attributed to the naiveté of native prey–an inability to detect novel predators as a threat and thus a failure to flee or defend themselves [2]. However, naiveté is unlikely to be an ‘all-or-nothing’ state [3], as prey may learn [4,5] and/or adapt over evolutionary time [6], as experience with predators increases [7]. Initially, introduced predators may have unfamiliar hunting techniques [3], or cues (e.g. their odour), that might not be recognised by local prey (e.g. [8]). This can lead to early and rapid impact of introduced predators. For example, prey on predator-free islands or in locations with only certain predator ‘groups’ (e.g. raptors or reptiles)[2] have been particularly badly affected by alien predators (e.g. [9,10]). Failure to recognise predation risk is a prerequisite for “island syndrome” or level 1 naiveté [7] where no anti-predator behaviour is displayed. However, no predator can remain eternally novel and in time, fauna that persist should lose their naiveté [3]. There is indirect support for this idea. For example, Pacific island skinks (*Caledoniscincus austrocaledonicus*) respond to odour cues of Pacific rats (*Rattus exulans*) with which they have experience for thousands of years, but not to odour from ship rats (*Rattus rattus*) or feral cats (*Felis catus*), to which they have been exposed for less than 300 years [11]. However, identifying actual changes in naiveté is difficult as studies are generally conducted at only one time since invasion (e.g. [8]) or via a comparison of prey responses to different predators with different invasion histories (e.g. [11]).

In this study we test a prediction of the hypothesis that naiveté can be overcome by examining responses of Australian native bandicoots (Family Peramelidae) towards introduced dogs and cats, with which they have co-existed for approximately 200 years [12,13]. Australia is an island continent which–until a few thousand years ago–had no large placental predators [13]. The first one was the dingo (*Canis lupus dingo*) which arrived on mainland Australia approximately 4000 years ago, entering via northern Australia [14]. The dingo is thought at least partially responsible for the extinction of mainland populations of Tasmanian devils (*Sarcophilus harrisii*), thylacines (*Thylacinus cynocephalus*) and the Tasmanian native hen (*Tribonyx mortieri*) [15]. Although dingoes meet many definitions of an alien species (having been transported by humans to a new location- sensu Richardson et al. [16]), many consider dingoes to be a native species in Australia. For example, according to the Australian federal legislation, species which arrived prior to 1400 (including the dingo) are considered a native species [17,18]. In contrast, cats are considered an alien species, because they have only been in Australia since Europeans arrived, ca. 200 years ago [12].

Carthey and Banks [19] suggested a new criterion for native status for naturalised species such as the dingo—that native species recognise them as predators. They used a novel approach of surveying residents in peri-urban Sydney about sightings and activity of long-nosed (*Perameles nasuta*) and southern brown (*Isoodon obesulus*) bandicoots in their backyards in relation to ownership of domestic dogs *C*. *lupus familiaris* (a close relative of the *C*. *lupus dingo*) and cats. Bandicoots in this study avoided backyards with dogs and several residents reported their dogs had killed bandicoots. The avoidance behaviour was hypothesised to represent not only recognition of dogs as predators but also an effective response, and support for the idea that the once alien dingo should be considered native (Carthey and Banks 2012). Notably though, bandicoots did not avoid yards with cats, which the authors hypothesised may indicate that 200 years is insufficient experience for anti-predator tactics to develop in bandicoots.

If bandicoot avoidance of dogs is due to prior exposure to dingoes then we predicted that bandicoots without prolonged coexistence with dingoes should remain naïve toward all dogs. We tested this prediction by taking advantage of the absence of dingoes in Tasmania, Australia’s largest offshore island, which dingoes never reached. However, domestic dogs were introduced to Tasmania by the first European settlers at the beginning of the 19th century, and are thought responsible for the loss of the Tasmanian emu (*Dromaius novaehollandiae diemensis*) and a 90% reduction of the range of the Forester kangaroo (*Macropus giganteus tasmaniensis*) [20,21]. Nevertheless, many species of wildlife extinct on the mainland persist in Tasmania [22].

Alternatively, it is possible that the responses of bandicoots to dogs observed by Carthey and Banks (2012) is due to a form of preadaptation resulting from experiences with native (marsupial) predators. Mainland and Tasmanian bandicoots have evolved with a range of native marsupial predators, including the Tasmanian devil [23,24] and several species of quolls (e.g. Eastern quolls *Dasyurus viverrinus*, [25]). Both groups of bandicoots also evolved with raptors, owls [26–28] and large reptiles [29]. Aborigines also hunted bandicoots for food and some still do in northern Australia [30–32]. Bandicoots’ shared evolutionary history with a range of native predators means we would not necessarily expect them to display “island syndrome”, or a complete failure to recognise any predation risk [2].

To distinguish between these two scenarios, we conducted a citizen science project within and around Hobart (postcodes 7000–7055), the largest city of Tasmania, following methods used by Carthey and Banks (2012) in Sydney, the largest city of mainland Australia. We surveyed urban and peri-urban Hobart residents to determine whether Tasmanian bandicoots would avoid backyards of dog owners more than yards of residents who did not own cats or either pet.

Sydney residents had two species of bandicoot potentially visiting backyards: southern brown and long-nosed bandicoots [33], although most reports were likely of long-nosed bandicoots. Hobart is also home to southern brown bandicoots, as well as eastern-barred bandicoots (*P*. *gunnii*). Both southern brown and eastern-barred bandicoots are closely related to long-nosed bandicoots (all within the family Peramelidae), and listed as endangered in either all or parts of their range on the mainland [18]. Southern brown bandicoots are secure in Tasmania, whereas eastern-barred bandicoots are critically endangered on the mainland and declining in Tasmania [18]. The main threats for eastern-barred bandicoots are predation from introduced predators and in Tasmania also *Toxoplasmosis gondii* infection [18]. All species of bandicoots (Peramelidae) are within the critical weight range (35-5500g, [34]) and are within the prey range of cats and dogs [22].

If duration of experience (i.e., coexistence history) determines prey recognition of introduced predators, then we predict that, unlike bandicoots on mainland Australia, bandicoots in Tasmania where dogs are relatively recent (about 200 years ago) introductions should not avoid backyards with either dogs or cats.

## Materials and Methods

To compare bandicoot responses to backyard pets between mainland Australia and Tasmania, we surveyed residents using SurveyMonkey (www.surveymonkey.com). We modified the survey created by Carthey and Banks [19] to suit Hobart. This work was approved by the University of Tasmania’s Human Research Ethics Committee (HREC) (Approval number H0014287). Survey participants were self-selecting and the survey advertised via five public lectures, two radio interviews and advertisement in a local newspaper (The Mercury, 26/09/2014, p. 15) as well as local councils’ newsletters and a project Facebook page. Additionally, 1200 printed survey forms were delivered to randomly chosen areas of Hobart residents whose backyards backed onto bushland, because we expected those backyards were the most likely places for wildlife to visit.

We asked residents whether they had seen bandicoots or their droppings (scats), linking to a scat-identification guide created by Kasseen Cook for Forestry Tasmania stored on Dropbox (www.dropbox.com/s/h74zwxyrt0dpcf4/ScatIDSheets%20V2.ppt). Residents who had not encountered mammalian wildlife or their droppings were encouraged to fill in the survey, as were both, pet- and non-pet-owners. Unlike Carthey and Banks we did not ask the residents to record the muzzle-shaped *(or cone-shaped)* holes left by foraging bandicoots because they may have confused them with diggings of other animals that are common within Hobart such as echidnas (Michael Driessen, DPIPWE, pers. comm.). As in Carthey and Banks [19], we asked residents to consider their backyards only, as pets are rarely kept in front yards. We asked about the number of dogs and cats people owned and how frequently their pets were kept inside during the day and night, because bandicoots in Tasmania can also be active during the day [35]. We also added a new category for reports of roaming cats and dogs (as recommended by Michael Driessen, DPIPWE, pers. comm.), and sightings of cats belonging to neighbours. To control for other variables that might influence the presence or absence of bandicoots or their droppings in backyards, we included questions on backyard size, perceived accessibility of a backyard to wildlife, watering frequency, and garden features (abundance of shelter, native vegetation, planted fruits and vegetables, paving, provision of food or water, use of pesticides, herbicides or fungicides). The exact response options can be found in the data’s variable categories provided together with the data in the S1 File and the survey questionnaire in the S2 File.

We used Exact Tests to assess bandicoots’ use of accessible backyards with or without pets or roaming pets, according to the number of pets (i.e. one or multiple) present, and whether and when (day and/ or night) dogs or cats were kept inside. Odds-ratio assessment was conducted to examine effect sizes for seeing bandicoots or their scats in yards with or without cats or dogs. We also used Exact Tests to test whether other garden features predicted bandicoot presence or absence in backyards.

In cases where we could not conduct Exact Tests, we used chi-square tests (4 cases). To meet the assumptions for chi-square tests, cat ownership had to be simplified into three categories to overcome the problem of sparsely populated cells in the contingency table. These categories were 'no cats', 'neighbouring cats' and 'own cat(s)'—combining the 'one cat' and 'multiple cats' into one category. Respondents who answered 'don't know ' for the vegetation composition variable were removed from all analyses (i.e. garden variables vs bandicoot sightings/scats and garden variables vs pet ownership tests). NA's (resulting from no answer given or respondents entering NA or ‘not applicable’ themselves) were always excluded.

Adjusted standardised residuals (ASR’s) were calculated for contingency tables to determine where any detected differences between categories lay. ASR’s > I2I demonstrated a significant lack of fit of the null hypothesis [36], with positive ASRs indicating more respondents in a category than predicted by the null hypothesis, and negative ASRs indicating fewer respondents in a category than predicted by the null hypothesis. All analyses were conducted in IBM SPSS Statistics 22.0.

## Results

Neither the presence of dogs nor cats in backyards showed any relationship with bandicoot sightings or scats. This result did not change whether both or only one type (cat/dog) of pet was owned, or if roaming pets were included in the analysis (Table 1, Table A-E in S3 File). Similarly, keeping pets inside during the day or night did not affect whether bandicoots or their scats were sighted (Table 1, Table F-H in S3 File). There were no significant differences in the odds ratios for seeing bandicoots (cats: 0.76 (CI 0.5–116); dogs: 1.05 (CI 0.68–1.62)) or their scats (cats: 0.8 (CI 0.48–1.34); dogs: 1.46 (CI 0.87–2.45)) in yards with or without cats or dogs, indicating that effect sizes were not different from zero.

**Table 1:** Reports of bandicoot sightings or scats in regards to number of dogs/ cats, ownership of both pets, reports of roaming pets, or whether pets were kept inside at night or day. Results of the exact tests for each variable vs. each of the two measures (sightings and droppings) of bandicoot presence in backyards. Values are the exact probability (significance evaluated at α = 0.05) that each contingency table would occur if that particular combination of variables were independent.

Variable	Exact *p*	
	*Sightings*	*Scats*
Number of dogs	0.63	0.25
Number of cats	0.36	0.19
Pets and roaming pets	0.06	0.05
Dogs inside at day	0.21	0.21
Dogs inside at night	1	0.30
Cats inside at day	0.87	0.49
Cats inside at night	0.95	0.71

Our results are based on 548 residents who participated in the survey, of which 37% owned at least one dog and 20% owned at least one cat. Bandicoot casualties caused by cats were reported by 10 residents and caused by dogs by 14 residents. Three further records of bandicoot casualties were from people who owned both pet types and did not specify whether the cat(s) or dog(s) were responsible for the death.

Residents with backyards backing directly into bushland were most likely to report bandicoot sightings (p < 0.001, Table 2; 0–20 m: ASR = 6.2, Table A in S4 File) whereas those being further away than 500 m from bushland were less likely (ASR = -3.3, Table A in S4 File) to report them. The results were similar for the report of bandicoot scats (Table 2, p < 0.001), although reports were less likely at over 100 m away from bushland (ASR = - 2.2, Table A in S4 File). Consistent with the findings of Carthey and Banks (2012), bandicoot sightings and scats (p = < 0.001, p = 0.001 respectively, Table 2) were both more likely in larger backyards (Table B in S4 File). Bandicoot sightings and scats (p = 0.02, p = 0.003, respectively, Table 2, Table C in S4 File) were also higher in yards consisting mainly of garden/lawn (sightings: ASR = 2.6, droppings: ASR = 3.6) rather than mostly paved/tiled areas. Sightings (p < 0.001, Tables 1b and Table D in S4 File) and scats (p = 0.04, Tables 1b and Table D in S4 File) of bandicoots were also more likely in backyards with mainly native vegetation. The availability of vegetation cover also predicted bandicoot sightings and scats (p <0.001, p = 0.016, respectively, Tables 1b and Table E S4 File). Provision of drinking water was positively associated with bandicoot sightings and scats (p = 0.020, p = 0.043, respectively, Tables 1b and Table F in S4 File). Applying different plant watering regimes, growing fruit or vegetables, or using pesticides, herbicides or fungicides had no impact on the presence of bandicoot sightings or their scats (Table 2, Table G-K in S4 File).

**Table 2:** Reports of bandicoot sightings or scats in regards to backyard variables. Results of the exact tests for each variable vs. each of the two measures (sightings and droppings) of bandicoot presence in backyards. Values are the exact probability (significance evaluated at α = 0.05) that each contingency table would occur if that particular combination of variables were independent. Note, due to insufficient computer memory for the variable ‘Distance to bushland’ a Chi-square test was performed.

Variable	Exact/Chi-square *p*	
	*Sightings*	*Scats*
Distance to nearest bushland	<0.01	<0.01
Backyard size	<0.01	<0.01
More lawn than paved area	<0.01	0.03
Mostly native vegetation	<0.01	0.04
Vegetation shelter	<0.01	0.02
Drinking water provided	0.02	0.04
Plant watering regime	0.07	0.78
Vegetable/ fruit crops	0.31	0.34
Pesticides used	0.88	0.35
Herbicides used	0.44	0.08
Fungicides used	0.14	0.60

## Discussion

Our results support our hypothesis that bandicoots on mainland Australia recognise and respond to dogs as predators due to thousands of years of exposure to predation from dingoes. This is consistent with the idea that naiveté can be overcome with sufficient experience. We found that in Tasmania, where dingoes have never been present and dogs arrived as recently as cats as companion animals of the first settlers in the 19th century, bandicoots show no avoidance of either predator which contrasts with their responses to dogs on mainland Australia [19]. Predation from these predators was a real threat, with >25 residents reporting their pets had killed bandicoots. Yet the likelihood of seeing Tasmanian bandicoots or their scats was similar regardless of whether the backyards had dogs and/or cats. This result suggests that, unlike their mainland counterparts, Tasmanian bandicoots are naïve to the risks of predation by these predators and that introduced predator exposure for 200 years is insufficient for them to recognise and respond to a new predator. Moreover, our results do not support the notion that mainland bandicoots were somehow pre-adapted to recognise domestic dogs due to prior, evolutionary, exposure to marsupial predators.

Naiveté towards introduced predators comprises of multiple levels [7]. At level 1, the prey shows no recognition of the predation risk and displays no anti-predator behaviour. At level 2, the prey recognises the predator as a threat, but its behaviour is inappropriate for the hunting strategy of the predator. At level 3, the prey uses an appropriate anti-predator behaviour but the ‘superior hunting tactics’ of the predator lead nevertheless to the prey’s demise. Banks and Dickman [7] suggested that prey which survive the initial acute impacts of alien predators are likely to move through these levels as alien predators become more familiar to the local species. Our results support the idea that mainland bandicoots may have once showed level 1 naïveté towards dingoes, as Tasmanian bandicoots do for domestic dogs, but have since moved beyond this because we found that Tasmanian bandicoots did not respond to dog (or cat) presence.

At the individual level, prey animals may detect a predator but decide not to respond, if other factors such as sex, competition, sociality, hunger and disease outweigh the detected risk [37]. For example, a very hungry animal may choose to forage in the face of predation risk, whereas a satiated animal has more to lose if it were predated, and so will respond by avoiding the threat. An alternative hypothesis for our results would be that bandicoots in Hobart do recognise dogs, but continue to visit backyards because they are under energetic stress, or because the risks posed by dogs are outweighed by some benefit found in these yards. We did find that bandicoot visits were associated with larger yards, yards with more garden or lawn, yards with provided water, native vegetation and greater cover. Even if these features are sufficiently attractive to bandicoots that they overcome a fear of dogs and visit yards anyway, it is difficult to imagine why this might be the case for Hobart but not Sydney bandicoots. As a consequence, we do not believe this alternative hypothesis explains our results.

Prey can avoid predator encounters using many different information sources about predator presence, such as visual, auditory or olfactory cues, or indirect cues of risk such as safer denser habitat, to avoid exposure to predators. However, a direct predator sighting is the most immediate cue of threat and deeply ingrained [38]. For example, captive tammar wallabies from populations that have had 9500 years of isolation from any mammalian predator, attempted to escape from taxidermically mounted foxes [39] but not fox odour [40]. Reponses to direct predator presence however can be subtle. For example, fox squirrels *Sciurus niger* in Chicago do not stop handling food when a cat approaches, but will escape to safety once the approach distance becomes too close to the squirrel [41]. By trying to escape the moment a cat or dog attempts an attack can be risky, but the amount of risk can be reduced by keeping close to shelter which the predator cannot access. While it is possible that bandicoots used such subtle anti-predator tactics in Tasmania, it seems unlikely given that their mainland counterpart showed large scale avoidance of yards with dogs. Subtle tactics would also seem unsuccessful given the numbers of reports of pet attacks on wildlife that residents in our study reported. Moreover, we do not know for sure whether Tasmanian bandicoots failed to recognize predation threat by cats and dogs (level 1 naïveté in Banks and Dickman [7]), or whether their reactions were inappropriate (level 2 naïveté in Banks and Dickman [7]). However, what is evident is that their anti-predator responses are different and more obvious where they have had long-term exposure to a predator [7].

A key question emerging from our study is how long does it take for prey to overcome their initial naïveté? Our results, together with those of Carthey and Banks [19], suggest that on the mainland 4000 years of exposure to predators is long enough for recognition of predators to develop (given the different responses towards dogs). They also indicate that ca. 200 years of exposure to cats and dogs is not long enough for recognition to develop. Identifying how long it takes for prey to overcome naïveté matters because, we argue, this is central to predicting when species that were once alien might be considered native. Most studies of this issue have focussed on the re-emergence of anti-predator tactics after long-term isolation from predators (e.g. [42,43]) or studies of the responses of prey to predators with different periods of coexistence [44]. Both approaches are problematic as they do not allow predictions on how long it will take for prey to overcome naïveté towards novel predators. In any case each individual predator and prey system is likely to have species-specific traits that determine how long it takes for appropriate anti predator tactics to develop. Thus, instead of assuming naiveté has been overcome given an arbitrary time of exposure to an alien species, behavioural testing of prey responses are needed.

Our results also have implications for conservation practice in Tasmania. The apparent naïveté of Tasmanian bandicoots towards domestic animals as predators has significant implications for responsible pet ownership in Tasmania and other places, like islands where domestic animals are recent arrivals, i.e. a couple of centuries. For example, globally cats have contributed to over 8% of all bird, mammal and reptile extinctions and to declines of about 14% of critically endangered birds, mammals and reptiles on islands [9,45,46]. Domestic dogs can be disastrous to insular fauna that evolved in the absence of mammalian predators [47–50]. If cats and dogs are not recognised as a threat by native wildlife then wildlife are likely to be more vulnerable to cats and dogs than to native predators. Cats are a well-known predator of native wildlife throughout Australian cities [12,51], a fact acknowledged by many cat owners [52]. Naiveté towards cats by native wildlife using urban areas most likely exacerbates cat impacts. Data collected by Holderness-Roddam and McQuillan [22] on injured and killed native wildlife using direct observation and assessment by veterinarians has shown that dogs in Tasmania have a much more harmful effect on wildlife than domestic cats. This result was based on the fact that more attacks were recorded for dogs and larger dogs were a risk also for prey too large to be preyed on by cats [22].

As bandicoots seem to fail to recognise dogs and cats as a threat in Tasmania, it might be a surprise that bandicoots are able to persist within Hobart. Particularly, since there has been increasing evidence of both pets chasing or killing bandicoots [22]. Reasons for this might lie within the habitat heterogeneity, including open lawn areas adjacent to thick sheltering vegetation that Hobart provides not only at its urban bushland fringes and many reserves, but also in many residents’ gardens. This provides safe (shelter-abundant) and good quality feeding grounds for bandicoots. In our study, abundant shelter and a short distance to nearest bushland were the strongest predictors for reporting bandicoots in backyards. This importance of shelter has also been demonstrated in other wildlife studies looking at city gardens or greenspace (e.g. [53,54] and is well known for prey species in the wild [55–58]. However, it is possible that bandicoots attracted to backyards due to better foraging and shelter opportunities while unaware of the risk of dogs or cats present, may suffer high predation here (thus causing an ecological trap)–a hypothesis that needs testing in further research. Thus bandicoots are not necessarily currently safe in Hobart. The Eastern-barred bandicoot has been suffering severe declines in Tasmania between the 1950s-1990s [59] and the biggest threat has been predation by dogs and cats [18], and death through the parasite *Toxoplasmosis gondii* for which cats are the primary host [60,61].

## Conclusions

This study supports the hypothesis that bandicoots are still naïve to cats as well as dogs in Tasmania, where they were both introduced with the first European settlers about 200 years ago. Contrary to the mainland, where bandicoots avoided backyards of dog owners (in terms of lower activity), indicating that 4000 years of exposure to dingoes were enough to adapt to the threat of this once novel predator, neither dogs or cats affected the occurrence of bandicoots in Tasmanian backyards. However, abundant shelter within the backyard and proximity to native bushland were important predictors of bandicoot visitation. Thus, providing shelter and keeping native bushland patches rich in shelter for bandicoots is critical to protect bandicoots in Hobart.

## Supporting Information

S1 File Dataset. (XLSX)

S2 File Survey questionnaire. (PDF)

S3 File Table showing adjusted standardised residuals (ASRs) for cells of the contingency table comparing bandicoot sightings and scats in yards of A) dog ownership (number of dogs), B) cat ownership (number of cats), C) mixed pet ownership, D) pet ownership including reports of roaming (unowned) cats and dogs, E) frequency of dogs in backyards during the day, F) frequency of dogs in backyards during the night, G) frequency of cats in backyards during the day, and H) frequency of cats in backyards during the night.
(DOCX)

S4 File Table showing adjusted standardised residuals (ASRs) for cells of the contingency table comparing bandicoot sightings and Scats in yards of A) different distances to bushland (in m), B) different backyard sizes (in m^2^), C) backyard type, D) vegetation composition, E) vegetation ground cover, and F) fruit and vegetable crops, G) water provision, H) pesticide use, I) herbicide use, and J) fungicide use.
(DOCX)
