# Supplementary material for: Does Historical Coexistence with Dingoes Explain Current Avoidance of Domestic Dogs? Island Bandicoots Are Naïve to Dogs, unlike Their Mainland Counterparts
Source: PLoS One. 2016 Sep 7;11(9):e0161447. doi: 10.1371/journal.pone.0161447 (PMC5014422; doi:10.1371/journal.pone.0161447)
Supplement: S2 File — (PDF) [file pone.0161447.s002.pdf]

the Dr Edward Hall environment grants

The University of Sydney

UTAS

MACQUARIE UNIVERSITY

HOBART CITY COUNCIL

Be involved in a citizen science project on Hobart's wildlife in backyards –

How the public and scientists can work together to provide wildlife refuges in an urban area

Dear Hobart Resident,

You are invited to participate in a study on mammalian wildlife in Hobart's backyards. One aim is to work together to locate wildlife refuges within our city and to find out how we can provide more/ better refuges. To achieve that we also want to investigate the effects of cats and dogs on the presence of wildlife on private property. This research is part of a range of projects investigating whether Australian native mammals are naïve to the predation risks posed by introduced predators, such as cats and dogs. You were selected as a possible participant in this study because your property is adjacent to bushland known to harbor a variety of native mammals.

What you can do to help

Complete a 5-8 minute web-based questionnaire, comprising multiple-choice questions about cat- and dog-ownership and the frequency of wildlife visitations in your backyard, with opportunity for additional comments.

You may have noticed signs left behind by wildlife, like diggings or poo. Photographs of wildlife species mentioned in the survey can be found on the following website: www.ala.org.au/australias-species/ or www.tmag.tas.gov.au/visitor_information/maps and guides/field_guide and indirect signs can be found on the following link: www.dropbox.com/s/h74zwxyrt0dpcf4/ScatIDSheets%20V2.ppt. If you received this invitation in your mailbox, then you should find identification material in your letter. Even if you do not have dogs or cats or do not have a backyard accessible to wildlife, we are still interested in your response.

Confidentiality and Disclosure of Information

Any information that is obtained in connection with this study will remain confidential. Anything you fill into the questionnaire will be absolutely anonymous which means we will not know who you are and where exactly your property is. In the rare case that you might be identifiable by being likely to be unique in your area, for example by being an owner of an unusually large number of cats or dogs, your answers will remain confidential. We plan to publish the results in the peer reviewed literature for conservation biology. In any publication, information will be provided in such a way that you cannot be identified. Once published, a summary of the results will be made available on a public server, like SHaRED from TERN (see http://www.tern.org.au/SHaRED-and-share-alike-bgp3056.html for more information).

Your Consent

Your decision to participate or not will not prejudice your future relations with the University of Tasmania, Macquarie University or The University of Sydney. If you decide to participate, you are free to withdraw your consent and to discontinue participation at any time.

To do the Survey

If you would like to participate online, please go to the following web address: **www.surveymonkey.com/s/TVDFYG5** and click on the link at the top of the page to take you to the survey. Alternatively, you can call Dr Anke Frank (UTas), on 03 6226 2633 or +61 459035189 during business hours to complete the survey by telephone or just return the attached survey by mail to the address on the bottom of this page. The survey will close at midnight on the 31st March 2015.

Inquiries

Contact Dr Anke Frank (03 6226 2633) with any queries or questions. This study has been approved by the Tasmanian Social Sciences Human Research Ethics Committee. If you have concerns or complaints about the conduct of this study, please contact the Executive Officer of the HREC (Tasmania) Network on +61 3 6226 7479 or email human.ethics@utas.edu.au. Please quote ethics reference number [H0014287].

Please keep a copy of this information sheet if you choose to participate in the study.

Sincerely,
Dr Anke Frank
(on behalf of the wildlife in backyards research team)

Please return your survey forms to:
Dr Anke Frank
School of Biological Science | Private Bag 55 | University of Tasmania | Hobart 7001| Tasmania

Survey forms 'Backyard Wildlife Hobart'

Questions regarding the rough location of your place in relation to nearest bushland. Please enter your location details below. To keep your information anonymous, but for us to get a rough idea on where your place is, we would like to ask you for the following details:

1. **What is the name of the street/ road you are living in?**

____

2. **If you like, please indicate your location in terms of where within a house number range your place would be located (e.g. 1-10, 11, 20, 151-160, 1021-1030**).

   ____

3. **Do you have a house number that is …?**
   - ○ even (0, 2, 4, 6, 8 or ending on one of these numbers) or
   - ○ uneven (1, 3, 5, 7, 9 or ending on one of these numbers)

Comment: ____

4. **What is your Postcode (please enter a number between 7000 and 7055)?** __________

5. **What is your estimated distance of your backyard from the nearest native bushland?**
   - ○ 0-20 m
   - ○ larger than 20-50 m
   - ○ larger than 50-100 m
   - ○ larger than 100m – 500 m
   - ○ larger than 500m – 1000 m
   - ○ larger than 1000 m

Comment: ____

6. **What is the size of your backyard?**
   - ○ Don't have the slightest idea.
   - ○ less than 10 square meters
   - ○ 10-30 square meters
   - ○ 30-50 square meters
   - ○ 50-100 square meters
   - ○ more than 100 square meters

Comment: ____

7. **Is your backyard …?**
   - ○ Mostly paved/tiled
   - ○ Half garden/ lawn, and half pavement/ tiles
   - ○ Mostly garden/ lawn

Comment: ____

8. Is your garden vegetation…?

- Mainly Australian native plants (same as surrounding/ nearby bushland)
- Mainly Australian native plants, but distinct from surrounding/ nearby bushland
- Half native/ half non-native
- Mainly non-native
- Don't know

Comment: ____

9. Some animal species may be attracted to fruit and/or vegetable gardens. Do you grow…?

- Fruit
- Vegetables
- Both
- Neither

Comment: ____

10. Some species feel safer if there is dense vegetation cover to hide under. Dense vegetation cover that would conceal a wild animal from cats/ dogs in your backyard is

- N/A (no backyard)
- Absent
- Present, but only in one or a few little patches
- Present and abundant

Comment: ____

11. Trees (trunk diameter at least 10cm wide at breast eight) often provide escape options for wildlife. Does your backyard contain…?

- N/A (no backyard)
- No trees
- 1 tree
- 2-4 trees
- 5 or more trees

Comment: ____

12. Do you water your garden/ lawn…?

- N/A – I do not have a garden or lawn
- Daily
- Several times a week
- Once per week
- Once per fortnight
- Less than once per fortnight
- Never

Comment: ____

13. How confident are you to reliably distinguish between the following species of mammals?

To check your knowledge get the following open source App from the Tasmanian Museum and Art Gallery: www.tmag.tas.gov.au/visitor_information/maps_and_guides/field_guide or see the following link for pictures and descriptions of the species mentioned below: www.ala.org.au/australias-species/:

Please tick the relevant field:

Type of animal	Very confident	Somewhat confident	Not confident
**Rats and mice** (no matter whether native or introduced)			
**European rabbit**			
**Bandicoot** (no matter whether Southern brown or Eastern barred bandicoot)			
**Large Possum** (adult Brush-tail possum – at least cat-size)			
**Other Possum** (no matter whether Ringtail , Pygmy or young Brush-tail possum, or Sugar glider)			
**Little macropod** (cat-size or smaller than cat, no matter whether Pademelon /Bennett's Wallaby/ Potoroo or Bettong)			
**Large macropod** (larger than cat-size: Bennett's Wallaby or adult Pademelon)			
**Quolls** (no matter whether Eastern or Spotted-tailed)			
**Tasmanian Devil**			
**Echidna**			
**Platypus**			

14. If you like comment below which of these animals you are confident to identify to species level:

____

____

____

15. In your opinion, is your backyard accessible for wildlife…? Note: you can tick multiple

- that can climb
- that can jump over a low fence or hedge
- that can duck underneath a fence or squeeze through low gaps

Comment: ____

16. Do you own a dog?

- Yes, one
- Yes, multiple
- No

17. If you like, specify the breed of your dog(s) in this comments section: ______________________________

____

18. Is your dog…?

- N/A (don't have a dog)
- < 5 kg
- 5-10 kg
- 10-15 kg
- 15-30 kg
- > 30 kg

19. **If multiple dogs, please add weight of other dogs in comments section below**:

____

____

20. How often would your dog be in your backyard? Please answer for day time and night time.

Time of day	N/A (no dog)	Never	About once per month	About once per week	Several times per week	Always/ daily
Day						
Night						

Comment: ____

____

____

21. Do you own a cat that has access to your garden?

- Yes, one
- Yes, multiple
- No, I don't have a cat
- No, my cat stays solely indoors or is enclosed in a cat run/ enclosure outdoors
- No, but neighbours' or stray cats visit at least monthly

Comment: ____

____

22. If you have multiple cats, please enter the number of cats in the section here: _____________

23. How often would cat(s) be outside in your backyard?

Time of day	N/A (no dog)	Never	About once per month	About once per week	Several times per week	Always / daily
Day						
Night						

Comment: ____

____

24. Do you ever encounter cats or dogs in your backyard which you do not own?

	Cat	Cats	Dog	Dogs
No, never				
Yes, but rarely (about once in three months)				
Yes, frequently (more often than once in 3 months)				
Yes, often (at least once a week)				

Comment: ____

____

25. There are things that may attract or repel native and introduced mammals: Do you provide any of these potential attractants?
(Note: you can click one, both or neither answer)

- Water (e.g. pond, rain-drum, watering can, water bowl for pets, bird baths)
- Food (e.g. pet food, compost, bird seeds, scraps for chicken, accessible vegetable crops or fruit trees)

Comment: ____

26. Have you used any of the following potential repellents?

- Pesticides (e.g. rodent poison)
- Fungicides
- Herbicides

Comment: ____

27. If you own a dog or cat, has it ever killed any mammal that you know of?

- N/A (no pets)
- Prefer not to answer
- No, never
- Rarely
- Several times

28. Comment (describe which pet (cat or dog) killed what species – if known): ________________________________

____

____

____

____

____

____

____

____

____

____

____

____

____

____

____

____

____

29. Which mammal species have you seen in your garden within the last 3 months?

- None
- Rat
- Rabbit
- Bandicoot
- Large Possum
- Other possum
- Little macropod
- Large macropod
- Quoll
- Tasmanian Devil
- Echidna
- Platypus

Other (please specify): ______________________

Comment: ____

____

____

30. The presence of a species can often only be detected by the signs they leave behind, like droppings (see the following link for descriptions www.dropbox.com/s/h74zwxyrt0dpcf4/Scat IDSheets%20V2.ppt).

In the last 3 months, have you noticed droppings (poo) in your garden of one or more of the following species …?

- Rat
- Platypus
- Echidna
- Tasmanian Devil
- Quoll
- Large macropod
- Rabbit
- Bandicoot
- Large Possum
- Other possum
- Little Macropod
- Not sure
- Definitely not

Other (please specify): ____________________________

Comment: ____

____

____

31. If you have encountered droppings, how frequently do you encounter their droppings?

Frequency	Rat	Rabbit	Bandicoot	Possum	Little macropod
Always (daily)					
About once a week					
About once a month					
About once every couple of months					
Rarrely (e.g. once in 6 months or a year)					
Never					

Comment: ____

____

____

Please use this space, if you have any other comments:

Some backyard wildlife photos:

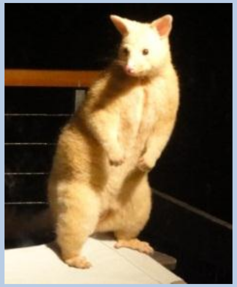

‘Blond’ brushtail possum

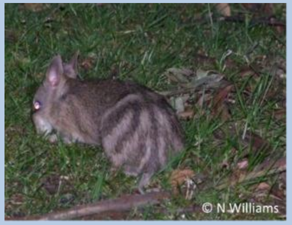

Eastern-barred bandicoot

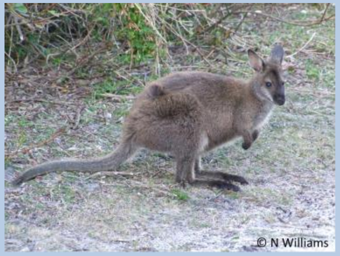

Bennet’s Wallaby

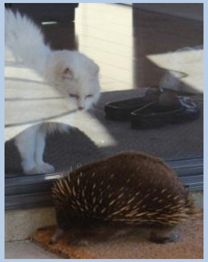

Echidna

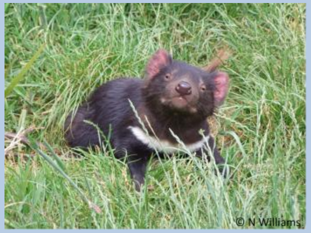

Tasmanian Devil

More Information on this project, including media coverage can be found on Facebook: www.facebook.com/pages/Backyard-wildlife-Hobart-a-citizen-science-project/
