# Supplementary material for: Does Historical Coexistence with Dingoes Explain Current Avoidance of Domestic Dogs? Island Bandicoots Are Naïve to Dogs, unlike Their Mainland Counterparts
Source: PLoS One. 2016 Sep 7;11(9):e0161447. doi: 10.1371/journal.pone.0161447 (PMC5014422; doi:10.1371/journal.pone.0161447)
Supplement: S3 File — Table showing adjusted standardised residuals (ASRs) for cells of the contingency table comparing bandicoot sightings and scats in yards of A) dog ownership (number of dogs), B) cat ownership (number of cats), C) mixed pet ownership, D) pet ownership including reports of roaming (unowned) cats and dogs, E) frequency of dogs in backyards during the day, F) frequency of dogs in backyards during the night, G) frequency of cats in backyards during the day, and H) frequency of cats in backyards during the night. (DOCX) [file pone.0161447.s003.docx]

**Table S1. Adjusted standardised residuals (ASRs) for cells of the contingency table comparing bandicoot sightings and scats in yards of A) dog ownership (number of dogs), B) cat ownership (number of cats), C) mixed pet ownership, D) pet ownership including reports of roaming (unowned) cats and dogs, E) frequency of dogs in backyards during the day , F) frequency of dogs in backyards during the night, G) frequency of cats in backyards during the day , and H) frequency of cats in backyards during the night.** ASRs greater than 2 indicate that more respondents chose that answer than predicted by the null hypothesis (no association between bandicoot sightings or Scats and distance to bushland), whereas ASRs less than -2 indicate the opposite. ‘na’ indicates that this category was never chosen by any participants in this study.

**A)**

|  | | **Dog ownership** | | | **Total responses** |
| --- | --- | --- | --- | --- | --- |
|  | | None | One | Multiple |  |
| Sightings | No  Yes | 0.6 | -1.0 | 0.4 | 240 |
|  |  | -0.6 | 1.0 | -0.4 | 148 |
| Scats | No | 1.7 | -1.2 | -0.9 | 312 |
|  | Yes | -1.7 | 1.2 | 0.9 | 76 |

**B)**

|  | | **Cat ownership** | | | | **Total responses** |
| --- | --- | --- | --- | --- | --- | --- |
|  | | None | Neighbours | One | Multiple |  |
| Sightings | No  Yes | -1.0 | 1.4 | -1.0 | 0.4 | 242 |
|  |  | 1.0 | -1.4 | 1.0 | -0.4 | 147 |
| Scats | No | -0.7 | 1.7 | -1.7 | 0.3 | 314 |
|  | Yes | 0.7 | -1.7 | 1.7 | -0.3 | 75 |

**C)**

|  | | **Mixed pet ownership** | | | | **Total responses** |
| --- | --- | --- | --- | --- | --- | --- |
|  | | No pets | Dog(s) only | Cat(s) only | Cat(s) and dog(s) |  |
| Sightings | No  Yes | -1.9 | 0.7 | 2.1 | -1.4 | 238 |
|  |  | 1.9 | -0.7 | -2.1 | 1.4 | 147 |
| Scats | No | -0.1 | -0.9 | 1.6 | -1.1 | 310 |
|  | Yes | 0.1 | 0.9 | -1.6 | 1.1 | 75 |

**D)**

|  | | **Including roaming pets** | | | |  |
| --- | --- | --- | --- | --- | --- | --- |
|  | | No pets owned or frequently roaming | Dog(s) only owned or frequently roaming | Cat(s) only owned or frequently roaming | Cat(s) and dog(s) owned or frequently roaming |  |
| Sightings | No  Yes | -1.4 | 0.8 | 2.0 | -1.9 | 238 |
|  |  | 1.4 | -0.8 | -2.0 | 1.9 | 147 |
| Scats | No | 0.8 | -0.5 | 1.9 | -2.5 | 310 |
|  | Yes | -0.8 | 0.5 | -1.9 | 2.5 | 75 |

**E)**

|  | |  | **Dog(s) in backyard during the day** | | | | **Total responses** |
| --- | --- | --- | --- | --- | --- | --- | --- |
|  | | Never | About once per month | About once per week | Several times per week | Always |  |
| Sightings | No  Yes | -1.4 | 1.1 | na | -1.1 | 1.4 | 80 |
|  |  | 1.4 | -1.1 | na | 1.1 | -1.4 | 51 |
| Scats | No | 1.4 | 0.8 | na | -1.4 | 0.3 | 101 |
|  | Yes | -1.4 | -0.8 | na | 1.4 | -0.3 | 30 |

**F)**

|  | | **Dog(s) in backyard during the night** | | | | | **Total responses** |
| --- | --- | --- | --- | --- | --- | --- | --- |
|  | | Never | About once per month | About once per week | Several times per week | Always |  |
| Sightings | No  Yes | -1.4 | -0.1 | na | 0.3 | 1.2 | 71 |
|  |  | 1.4 | 0.1 | na | -0.3 | -1.2 | 43 |
| Scats | No | -1.1 | 0.2 | na | -1.0 | 1.7 | 87 |
|  | Yes | 1.1 | -0.2 | na | 1.0 | -1.7 | 26 |

**G)**

|  | | **Cat(s) in backyard during the day** | | | | | **Total responses** |
| --- | --- | --- | --- | --- | --- | --- | --- |
|  | | Never | About once per month | About once per week | Several times per week | Always |  |
| Sightings | No  Yes | 0.1 | 1.0 | -0.5 | -0.4 | -0.3 | 75 |
|  |  | -0.1 | -1.0 | 0.5 | 0.4 | 0.3 | 47 |
| Scats | No | 0.3 | 1.5 | -0.2 | 0.7 | -1.6 | 96 |
|  | Yes | -0.3 | -1.5 | 0.2 | -0.7 | 1.6 | 26 |

**H)**

|  | | **Cat(s) in backyard during the night** | | | | | **Total responses** |
| --- | --- | --- | --- | --- | --- | --- | --- |
|  | | Never | About once per month | About once per week | Several times per week | Always |  |
| Sightings | No  Yes | -0.5 | -0.1 | 0.8 | 0.0 | 0.0 | 71 |
|  |  | 0.5 | 0.1 | -0.8 | 0.0 | 0.0 | 40 |
| Scats | No | 0.0 | 0.3 | 0.9 | 0.4 | -1.3 | 91 |
|  | Yes | 0.0 | -0.3 | -0.9 | -0.4 | 1.3 | 20 |
