# Supplementary material for: Does Historical Coexistence with Dingoes Explain Current Avoidance of Domestic Dogs? Island Bandicoots Are Naïve to Dogs, unlike Their Mainland Counterparts
Source: PLoS One. 2016 Sep 7;11(9):e0161447. doi: 10.1371/journal.pone.0161447 (PMC5014422; doi:10.1371/journal.pone.0161447)
Supplement: S4 File — Table showing adjusted standardised residuals (ASRs) for cells of the contingency table comparing bandicoot sightings and Scats in yards of A) different distances to bushland (in m), B) different backyard sizes (in m2), C) backyard type, D) vegetation composition, E) vegetation ground cover, and F) fruit and vegetable crops, G) water provision, H) pesticide use, I) herbicide use, and J) fungicide use. (DOCX) [file pone.0161447.s004.docx]

**S4 File Tables.** Adjusted standardised residuals (ASRs) for cells of the contingency table comparing bandicoot sightings and Scats in yards of A) different distances to bushland (in m), B) different backyard sizes (in m^2^), C) backyard type, D) vegetation composition, E) vegetation ground cover, and F) fruit and vegetable crops, G) water provision, H) pesticide use, I) herbicide use, and J) fungicide use. ASRs greater than 2 indicate that more respondents chose that answer than predicted by the null hypothesis (no association between bandicoot sightings or Scats and distance to bushland), whereas ASRs less than -2 indicate the opposite.

**A)**

|  | | **Distance to bushland (m)** | | | | | | **Total responses** |
| --- | --- | --- | --- | --- | --- | --- | --- | --- |
|  | | 0-20 | 20-50 | 50-100 | 100-500 | 500-1000 | >1000 |  |
| Sightings | No  Yes | -6.2 | 0.6 | 1.0 | 1.9 | 3.3 | 3.2 | 241 |
|  |  | 6.2 | -0.6 | -1.0 | -1.9 | -3.3 | -3.2 | 147 |
| Scats | No | -3.4 | -1.0 | 0.9 | 2.2 | 1.6 | 1.8 | 313 |
|  | Yes | 3.4 | 1.0 | -0.9 | -2.2 | -1.6 | -1.8 | 75 |

**B)**

|  | | **Backyard size (m^2^)** | | | | | **Total responses** |
| --- | --- | --- | --- | --- | --- | --- | --- |
|  | | <10 | 10-30 | 30-50 | 50-100 | >100 |  |
| Sightings | No  Yes | 1.9 | 2.8 | 2.4 | 2.3 | -5.8 | 221 |
|  |  | -1.9 | -2.8 | -2.4 | -2.3 | 5.8 | 136 |
| Scats | No | 0.8 | 2.0 | 2.0 | 1.9 | -4.4 | 286 |
|  | Yes | -0.8 | -2.0 | -2.0 | -1.9 | 4.4 | 71 |

**C)**

|  | | **Backyard type** | | | **Total responses** |
| --- | --- | --- | --- | --- | --- |
|  | | Mostly paved/ tiled | Half garden/ lawn, half paved/ tiled | Mostly garden/ lawn |  |
| Sightings | No  Yes | 1.5 | 3.1 | -3.5 | 233 |
|  |  | -1.5 | -3.1 | 3.5 | 188 |
| Scats | No | 1.0 | 2.4 | -2.6 | 300 |
|  | Yes | -1.0 | -2.4 | 2.6 | 71 |

**D)**

|  | | **Vegetation composition** | | | | **Total responses** | |
| --- | --- | --- | --- | --- | --- | --- | --- |
|  | | Mainly non-native | Half native/ half non-native | Mainly Australian native but distinct from surrounding/ nearby bush | Mainly Australian native plants which are the same as surrounding/ nearby bush | |  |
| Sightings | No  Yes | 4.0 | -0.1 | -1.2 | -3.0 | | 240 |
|  |  | -4.0 | 0.1 | 1.2 | 3.0 | | 145 |
| Scats | No | 2.3 | -0.1 | 0.0 | -2.3 | | 310 |
|  | Yes | -2.3 | 0.1 | 0.0 | 2.3 | | 75 |

**E)**

|  | | **Vegetation ground cover** | | | **Total responses** |
| --- | --- | --- | --- | --- | --- |
|  | | Absent | Present but only in one or a few little patches | Present and abundant |  |
| Sightings | No  Yes | 1.4 | 3.3 | -4.3 | 240 |
|  |  | -1.4 | -3.3 | 4.3 | 147 |
| Scats | No | 1.8 | 1.5 | -2.6 | 312 |
|  | Yes | -1.8 | -1.5 | 2.6 | 75 |

**F)**

|  | | **Water provision** | | **Total responses** |
| --- | --- | --- | --- | --- |
|  | | No | Yes |  |
| Sightings | No  Yes | 2.4 | -2.4 | 244 |
|  |  | -2.4 | 2.4 | 148 |
| Scats | No | 2.1 | -2.1 | 316 |
|  | Yes | -2.1 | 2.1 | 76 |

**G)**

|  | | **Watering regime** | | | | | **Total responses** |
| --- | --- | --- | --- | --- | --- | --- | --- |
|  | | Never | Less than once per fortnight | Once per fortnight | Once per week | Daily |  |
| Sightings | No  Yes | -1.0 | -0.1 | -1.8 | 2.4 | 0.8 | 184 |
|  |  | 1.0 | 0.1 | 1.8 | -2.4 | -0.8 | 114 |
| Scats | No | 1.0 | -0.1 | 0.2 | 0.7 | 0.9 | 241 |
|  | Yes | -1.0 | 0.1 | -0.2 | -0.7 | -0.9 | 57 |

|  |  |  |  |  |  |  |  |  |
| --- | --- | --- | --- | --- | --- | --- | --- | --- |

**H)**

|  | | **Fruit and vegetable crops** | | | |  |
| --- | --- | --- | --- | --- | --- | --- |
|  | | Neither, nor fruit or vegetables | Fruit only | Vegetable only | Both, fruit and vegetables |  |
| Sightings | No  Yes | 0.9 | -0.2 | 1.5 | -1.5 | 244 |
|  |  | -0.9 | 0.2 | -1.5 | 1.5 | 146 |
| Scats | No | 1.5 | -1.0 | 0.8 | 0.0 | 314 |
|  | Yes | -1.5 | 1.0 | -0.8 | 0.0 | 76 |

**I)**

|  | | **Pesticide use** | | **Total responses** |
| --- | --- | --- | --- | --- |
|  | | No | Yes |  |
| Sightings | No  Yes | -0.2 | 0.2 | 244 |
|  |  | 0.2 | -0.2 | 148 |
| Scats | No | 1.1 | -1.1 | 316 |
|  | Yes | -1.1 | 1.1 | 76 |

**J)**

|  | | **Herbicide use** | | **Total responses** |
| --- | --- | --- | --- | --- |
|  | | No | Yes |  |
| Sightings | No  Yes | 0.8 | -0.8 | 244 |
|  |  | -0.8 | 0.8 | 148 |
| Scats | No | 1.8 | -1.8 | 316 |
|  | Yes | -1.8 | 1.8 | 76 |

**K)**

|  | | **Fungicide use** | | **Total responses** |
| --- | --- | --- | --- | --- |
|  | | No | Yes |  |
| Sightings | No  Yes | 1.5 | -1.5 | 244 |
|  |  | -1.5 | 1.5 | 148 |
| Scats | No | 0.6 | -0.6 | 316 |
|  | Yes | -0.6 | 0.6 | 76 |
